# Adult-onset acute disseminated encephalomyelitis mimicking complex migraine: A CARE-compliant case report

**DOI:** 10.1097/MD.0000000000042458

**Published:** 2025-05-16

**Authors:** Abdallah A. Najjar, Mahmoud A. Abu Mayaleh, Roaa M. Halaykh, Majd Y. Amleh, Saeed I. Atawnah

**Affiliations:** a Hebron University, Hebron, Palestine; b Department of Internal Medicine, Al Ahli Hospital, Hebron, Palestine.

**Keywords:** ADEM, adult-onset ADEM, central nervous system disorder, demyelinating disorders, migraine, neurology case report

## Abstract

**Rationale:**

Acute disseminated encephalomyelitis (ADEM) is an immune-mediated demyelinating disorder of the central nervous system, often triggered by infection or vaccination. It is rare in adults and can mimic other conditions, including complex migraine, leading to potential misdiagnosis and delayed treatment.

**Patient concerns:**

A 35-year-old male presented with a progressive course of headache, visual disturbances, and altered mental status after an initial diagnosis of migraine.

**Diagnoses:**

Initial imaging was nonspecific; however, repeat magnetic resonance imaging showed multifocal high T2-weighted fluid-attenuated inversion recovery signal intensities. Cerebrospinal fluid analysis revealed elevated protein and lymphocytic pleocytosis. Infectious and autoimmune causes were excluded, supporting a diagnosis of ADEM.

**Interventions:**

The patient was treated with high-dose intravenous methylprednisolone followed by a tapering course of oral corticosteroids. Supportive therapy included antipsychotics and anticonvulsants.

**Outcomes:**

Substantial clinical improvement was noted, including restoration of orientation and motor function. At the 2-week follow-up, the patient remained neurologically stable.

**Lessons:**

This case emphasizes the importance of considering ADEM in adults with atypical migraine presentations. Early recognition and corticosteroid therapy can lead to favorable outcomes.

## 1. Introduction

Acute disseminated encephalomyelitis (ADEM) is an immune-mediated demyelinating syndrome of the central nervous system abbreviated as ADS, mostly seen in children. It is defined by hemisphere psoriasis in most cases, more or less in demyelination even in the white matter following an infection or infrequently, a vaccine. It is tentatively believed that a classic T cell autoimmune response against myelin basic polypeptide, which disrupts the normal functioning of the central nervous system, is responsible for the condition.^[1,2]^

Clinically, ADEM presents as a single acute episode with multifocal symptoms, including motor, sensory, and cranial nerve deficits, brainstem impairment, altered mental status, and optic neuritis. Encephalopathy frequently presents with similar signs and symptoms and therefore serves as a distinguishing characteristic that makes ADEM a unique disorder from other demyelinating disorders.^[3,4]^ Diagnostic evaluations typically include cerebrospinal fluid (CSF) analysis, lymphocytic pleocytosis and elevated protein levels, and magnetic resonance imaging (MRI), which shows characteristic bilateral, asymmetric white matter lesions. Initial treatment generally involves high-dose intravenous corticosteroids for 3 to 5 days, with intravenous immunoglobulin (IVIG) or plasma exchange considered in refractory cases. Acyclovir is often initiated empirically to exclude a viral etiology until further testing is complete.^[2,5]^

ADEM is responsible for approximately 22% to 32% of pediatric ADS cases with the majority of the cases appearing as monophasic type. However, the clinical overlap with other demyelinating conditions, such as clinically isolated syndrome, multiple sclerosis, or neuromyelitis optica spectrum disorder, can cause diagnostic challenges.^[3,6]^ Specifically, the determination of myelin oligodendrocyte glycoprotein (MOG)-antibodies has risen as a relevant biomarker to help discriminate ADEM from other ADSs.

MOG-antibodies, which target the central nervous system-specific MOG protein, have been associated with age-dependent disease phenotypes, including ADEM in younger children and optic neuritis or myelitis in older individuals.^[6,7]^

Moreover, ADEM may present mimicking other conditions, such as migraine, especially in pediatric patients. Overlapped symptoms, including headache, visual disturbances, and nausea, all of that may delay the diagnosis, especially at early stages when the neurological findings are subtle or absent.^[8,9]^ The overlap accentuates the need for broader clinical and imaging assessments so as to make an accurate diagnosis and commence treatment early in the process.

## 2. Case presentation

A 35-year-old male was presented with a 12-hour history of decreased consciousness following a complex progression of symptoms over the preceding week. The patient’s baseline health was characterized by chronic insomnia, persistent fatigue, and emotional stress, which had impacted his daily functioning. One week prior to admission, he began experiencing severe headaches, generalized joint pain (arthralgias), and muscle aches (myalgias). Seeking medical attention, he was initially evaluated at a local hospital where he was diagnosed with an upper respiratory tract infection and treated with intravenous fluids, resulting in temporary symptomatic relief.

He was reevaluated at the same hospital and presented with a severe, throbbing headache, visual disturbances, and sensitivity to light and sound-symptoms that were highly suggestive of a complex migraine. Over-the-counter treatments provided minimal relief, prompting further evaluation. Neurological examination was unremarkable, and a brain MRI was performed, revealing nonspecific findings typically associated with migraine. Given the combination of symptoms and imaging results, the patient was diagnosed with complex migraine and discharged. The patient was reassured and discharged home, where he noted a temporary improvement.

Despite initial improvement, the patient developed further concerning symptoms, such as difficulty sleeping, worsening headaches, tongue heaviness, hypoactivity, numbness in the right hand, and bluish discoloration of the lips.

However, within days, his symptoms recurred and progressively worsened, prompting multiple visits to healthcare facilities. During these visits, his condition worsened with new symptoms, including speech difficulties, numbness in his right hand, and episodes of disorientation. He was found to have hypotension but declined further intravenous fluid therapy. In the following days, his condition deteriorated further, presenting with new symptoms such as speech difficulties, numbness in his right hand, and episodes of disorientation. These developments, particularly the motor and sensory deficits, were atypical for migraine and suggested a more serious underlying pathology.

On the day of admission to our hospital, he had a temperature of 100.76F (38.2°C), heart rate of 104 beats/min, blood pressure of 133/80 mm Hg, and oxygen saturation of 95%. He was deteriorated significantly, became hypoactive, confused, unaware of his surroundings, and exhibited abnormal behavior with poor responsiveness. His family, who provided the history, reported that he had been increasingly disoriented and unresponsive since the early morning hours. Considering the rapid progression of symptoms, he was brought to the hospital for further evaluation and management.

Upon examination, the patient was found to be in a state of confusion, with a Glasgow Coma Scale score of 10/15. Physical examination revealed swelling in the right eye, tachycardia, and decreased breath sounds on chest auscultation. Neurological examination was challenging due to the patient’s agitation, but it was noted that he responded to painful stimuli, particularly when his neck was moved, suggesting possible meningeal irritation. Initial imaging with a brain CT scan and limited brain MRI did not reveal acute ischemic changes or intracranial hemorrhage.

**Figure 1.**
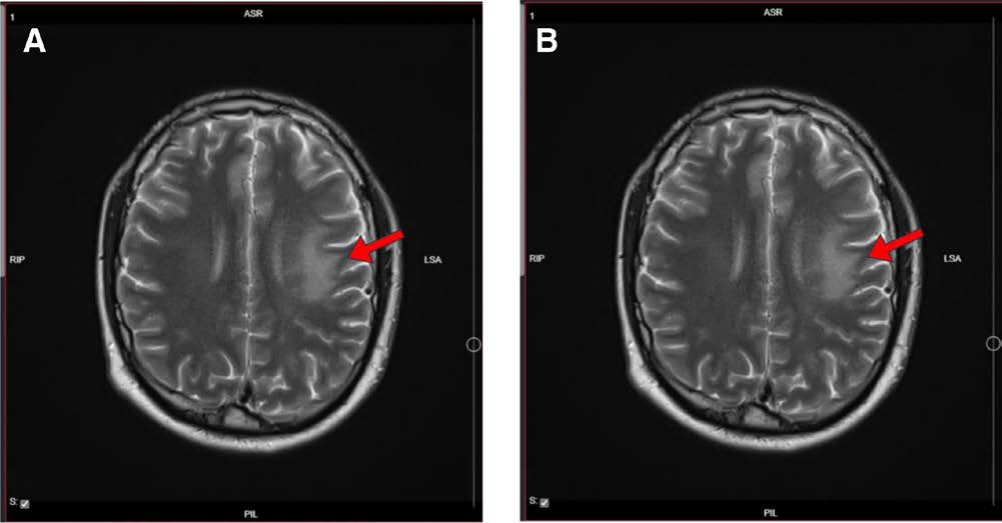
(A) T2-weighted MRI showing hyperintense lesions in the periventricular and subcortical white matter, consistent with areas of active inflammation and demyelination seen in ADEM. The red arrow indicates one of these lesions. (B) FLAIR MRI showing the same hyperintense lesions, one of which is indicated by the red arrow, with better delineation due to the suppression of the CSF background. ADEM = acute disseminated encephalomyelitis, CSF = cerebrospinal fluid, FLAIR = fluid attenuated inversion recovery.

According to the inconclusive initial imaging and the severity of the clinical presentation, A follow-up brain MRI was performed, which revealed newly developed areas of high T2-weighted fluid attenuated inversion recovery signal intensities in the left hemisphere, right parietal, and temporal lobes, as well as mild leptomeningeal enhancement (Fig. 1). Extensive laboratory investigations including a hematological workup (Table 1), basic metabolic and liver panel (Table 2), CSF analysis (Table 3), and drug toxicology screen (Table 4) were performed. CSF analysis revealed elevated protein levels and lymphocytic pleocytosis, supporting the diagnosis of ADEM. PCR testing for viral pathogens, including HSV, was negative. Given the absence of bacterial growth in CSF cultures and negative PCR results, empiric antiviral and antibiotic therapies were discontinued after 5 days.

**Table 1:** Hematology results.

Test	Result
White blood cell count (×10^3^)	13.38
Red blood cell count (×10^6^/µL)	5.629
Hemoglobin	15.6
Hematocrit	47.81
Mean corpuscular volume (µm^3^)	84.935
Mean corpuscular hemoglobin (pg)	27.714
Mean corpuscular hemoglobin concentration (g/dL)	32.629
Platelet count (×10^3^/µL)	320.1
Neutrophils (%)	61.3
Absolute neutrophils (×10^3^/µL)	8.214
Lymphocytes (%)	28.5
Absolute lymphocytes (×10^3^/µL)	3.824
Monocytes (%)	7.7
Absolute monocytes (×10^3^/µL)	1.031
Eosinophils (%)	1.33
Absolute eosinophils (×10^3^/µL)	0.179
Basophils (%)	0.984
Absolute basophils (×10^3^/µL)	0.132
RDW	12.16

RDW = red cell distribution width.

**Table 2:** Clinical chemistry.

Test	Result
Calcium (mg/dL)	9.6
Albumin, S (g/dL)	4.8
Sodium (Na) (mEq/L)	135
Potassium (K) (mEq/L)	4.1
Chloride (mmol/L)	104
Blood urea nitrogen (mg/dL)	15
Creatinine (mg/dL)	0.99
Alanine aminotransferase (U/L)	22
Aspartate aminotransferase (U/L)	20
Bilirubin, T (mg/dL)	0.7
Bilirubin, D (mg/dL)	0.3
Alkaline phosphatase (U/L)	62
RBS	96
Magnesium (Mg) (mg/dL)	2.34
Phosphorus (mg/dL)	4.4

**Table 3:** CSF analysis.

Test	Result
Tubes	4 × 1 mL
Color	Watery
Turbidity	Slightly turbid
Leukocytes	750
Erythrocytes	30
Neutrophils (%)	10
Lymphocytes (%)	70
Monocytes (%)	20
Plasma cells	NA
Other cells	NA
Gram stain	NA
CSF protein (mg/dL)	145
CSF glucose (mg/dL)	52
Glucose/serum	NA
Lactate dehydrogenase/fluid	NA
Xanthochromia	NA

CSF = cerebrospinal fluid, NA = not available.

**Table 4:** Drug/toxicology screening.

Drug	Result
Amphetamine	Negative
Cocaine	Negative
Morphine	Negative
Cannabis	Negative
Mephitamine	Negative
Benzodiazepine	Negative
Methadone	Negative
Barbiturate	Negative
Ecstasy	Negative
Tricyclic antidepressants	Negative

The patient was promptly started on high-dose intravenous methylprednisolone as part of a pulse steroid therapy regimen. Over the course of 3 days, his level of consciousness and orientation improved significantly. However, he continued to experience episodes of agitation, for which benzodiazepines were administered as needed. The treatment plan included tapering the steroids over 4 to 6 weeks, transitioning to oral prednisolone 40 mg 1 × 1. An antipsychotic (Haloperidol) 5 mg 3× PRN was prescribed to manage agitation, and anticonvulsant therapy was initiated for seizure prophylaxis.

Throughout his hospital stay, the patient was closely monitored for complications. A single episode of urinary incontinence was documented, but there were no other significant adverse events. At the time of discharge, the patient had returned to full orientation, with resolution of confusion and marked improvement in cognitive and motor functions. He was able to walk unassisted, follow commands, and perform activities of daily living independently. Mild residual fatigue and occasional mood fluctuations were reported but did not interfere with his functional status. The discharge diagnosis was ADEM, most likely triggered by a recent viral illness. A follow-up MRI was scheduled, and outpatient neurology visits were arranged for continued monitoring. At the 2-week follow-up, the patient remained clinically stable without recurrence of neurological symptoms, suggesting a favorable short-term prognosis.

### 2.1. Ethical approval

Ethical approval was waived for this single-patient case report, as it did not involve research requiring institutional review board oversight. Written informed consent was obtained from the patient for publication of this case report and accompanying images.

## 3. Discussion

This case highlights that it is crucial to carry out a comprehensive history and physical examination to distinguish between migraine symptoms and other neurological conditions.

Initially, the patient was diagnosed with an upper respiratory tract infection but due to the worsening headache and brain MRI findings, the patient was diagnosed with migraine. However, after a few days due to the progression of his symptoms, he was admitted to our hospital and presented with fever, confusion, hypoactivity, abnormal behavior, and poor response, the MRI was repeated and according to its findings, the migraine was excluded. PCR was done excluding the most common viral pathogens and CSF cultures were negatives for bacterial growth. Also, Bechet disease was one of the deferential diagnoses, so the Pathergy test and Eye exam were done and both showed normal results so this diagnosis was excluded, and ADEM is suspected. His brain MRI showed areas of high T2-weighted fluid attenuated inversion recovery signal intensities in the left hemisphere, right parietal, and temporal lobes, as well as mild leptomeningeal enhancement which makes ADEM the only reasonable diagnosis.

ADEM is an acquired inflammatory demyelinating syndrome that causes an immune response to different types of infectious agents. The sequel of this overactive immune reaction can be life-threatening. It is predominately affecting the white matter of the brain and spinal cord in a multifocal distribution.^[10]^

It is often treated with high-dose intravenous corticosteroids as first-line therapy. One common protocol is 20 to 30 mg/kg/d of methylprednisolone (maximum dose of 1 g/d) for 3 to 5 days. Improvement may be observed within hours but usually requires several days. An oral taper for 4 to 6 weeks or some other interval is sometimes appended.

The chief alternative therapy is IVIG. It is administered as 2 g/kg intravenously over the course of 3 to 5 days.

In severe cases of ADEM, the combination of intravenous corticosteroids and IVIG is sometimes used; however, it is unclear if this approach confers any clinical advantage. Plasma exchange, either concurrent with or following high-dose steroids, is also a consideration in severe ADEM cases.

Severe and/or steroid-refractory ADEM often requires the use of second-line treatments such as cyclophosphamide or rituximab.^[11]^

## 4. Limitations

This case report has some limitations inherent to its nature. As a single-patient observation, the findings may not be generalizable to broader populations. Additionally, the lack of long-term follow-up data restricts assessment of delayed complications or recurrence. While the diagnosis of ADEM was supported by clinical presentation, imaging, and CSF analysis, testing for MOG and AQP4 antibodies was not available, which could have provided further diagnostic clarity. Despite these limitations, the case contributes valuable insight into the diagnostic process and therapeutic outcomes in adult-onset ADEM.

## 5. Conclusion

This case highlights the crucial need for comprehensive diagnostic assessment in patients with unusual neurological symptoms, especially if the initial diagnosis, like migraine, is inadequate. The progression of symptoms in our patient highlights the need for vigilance and the consideration of rare but significant conditions like ADEM in differential diagnoses. Recognizing and intervening promptly with high doses of corticosteroids led to marked clinical improvement, proving the efficacy of this treatment approach.

This report reminds clinicians to consider a wide range of possible diagnoses when dealing with changing or unusual clinical symptoms, so that patients receive timely and suitable treatment to avoid potential complications in the future.

## Acknowledgments

We would like to thank the patient and their family for participating in this study.

## Author contributions

**Conceptualization:** Abdallah A. Najjar, Mahmoud A. Abu Mayaleh, Roaa M. Halaykh, Majd Y. Amleh, Saeed Atawnah.

**Data curation:** Abdallah A. Najjar, Mahmoud A. Abu Mayaleh, Roaa M. Halaykh, Majd Y. Amleh, Saeed Atawnah.

**Formal analysis:** Abdallah A. Najjar, Mahmoud A. Abu Mayaleh, Roaa M. Halaykh, Majd Y. Amleh.

**Investigation:** Abdallah A. Najjar, Mahmoud A. Abu Mayaleh, Roaa M. Halaykh, Majd Y. Amleh, Saeed Atawnah.

**Methodology:** Abdallah A. Najjar, Mahmoud A. Abu Mayaleh, Roaa M. Halaykh, Majd Y. Amleh, Saeed Atawnah.

**Project administration:** Abdallah A. Najjar, Mahmoud A. Abu Mayaleh.

**Resources:** Abdallah A. Najjar, Mahmoud A. Abu Mayaleh.

**Software:** Abdallah A. Najjar, Mahmoud A. Abu Mayaleh.

**Writing – original draft:** Abdallah A. Najjar, Mahmoud A. Abu Mayaleh, Roaa M. Halaykh, Majd Y. Amleh, Saeed Atawnah.

**Writing – review & editing:** Abdallah A. Najjar, Mahmoud A. Abu Mayaleh, Roaa M. Halaykh, Majd Y. Amleh, Saeed Atawnah.
